# Supplementary material for: Learning curve and surgical outcome of robotic assisted colorectal surgery with ERAS program
Source: Sci Rep. 2022 Nov 29;12:20566. doi: 10.1038/s41598-022-24665-w (PMC9709162; doi:10.1038/s41598-022-24665-w)
Supplement: Supplementary file 2 — Supplementary Information 2. [file 41598_2022_24665_MOESM2_ESM.zip › RAL Raw data-Ñ[▒K/126/OP N_ñw╝╨░O▒Kñσ.pdf]

W52 / W52

臺中榮民總醫院
手術室護理記錄

[redacted]

基本資料

- 室別： 開刀房-17室，手術科別： CRS，手術類別： 常規，日期： 2021/11/18
- 進入時間： 0730 ； 麻醉開始時間： 0757 ； Time Out時間： 0930 ； 劃刀時間： 0931 ； 手術結束時間： 1555 送出時間： 1620 ；
- 麻醉方式： GE

  診斷： Malignant tumor of rectum

  手術名稱： 1. Robotic (XI) assisted TME+ loop ileostomy
  2. Intraoperative colonoscopy : (-).
  3. High ligation (+) : At IMA root (Via robotic clips). IMV was ligated via endoclip.
  4. Take down from the splenic flexure : (-)
  5. Proximal cut end: > 10 cm, distal cut end: 1.5 cm
  6. No drainage tube was inserted
  7. The residual proximal D colon was sutured to distal rectal stump via hand sewn with 1-0 Vicryl
  8. Loop ileostomy (About 15~20cm from IC valve) was done over RLQ of abdomen

  手術局部用藥： nil

  術前預防性抗生素：
  - 抗生素名稱一： Veterin 1GM IVD/由麻醉護理人員給予
  - 時間： 0850
  - 上次簽章: 歐真勤
  - 抗生素名稱二： Veterin 1GM IVD/由麻醉護理人員給予
  - 時間： 1405
  - 記錄者： 廖子晴

  ，追加預防性抗生素：
- 手術醫師： 蔣鋒帆，陳瑢夫，蔡濬樟
- 麻醉醫師： VS 齊光彪
-
  - 刷手護士A ： 林貝臻，時間： 0730，刷手護士B ： 鄭鈞躍，時間： 0730，接替刷手一： 歐真勤，時間： 1200，接替刷手二： 林貝臻，時間： 1230
  - 流動護士A ： 歐真勤，時間： 0730，接替流動一： 鄭鈞躍，時間： 1130，接替流動二： 廖子晴，時間： 1230
- 更多接替及流動護士： 接替刷手三： 朱淨慧，時間： 1500，接替刷手四： 邱欐婇，時間： 1500，接替流動三： 劉雅汶，時間： 1500

皮膚狀況

A-V Shunt： 無

術前： 完整

記錄者： 上次簽章: 歐真勤

術後： 完整

記錄者： 上次簽章: 劉雅汶

手術準備

手術臥式 單一手術臥式

-
  - 手術臥式： 截石臥式
  - 使用附件： 溫毯 38 ℃，頭圈，腳架
  - 皮膚準備： 10%Aq Betadine，Hibiscrub，2% Chlorhexidine
  - 電燒： 使用電燒: 電極片位置： 其它： 導電軟墊，雙極電燒，記錄者： 上次簽章: 歐真勤

止血帶T： 無

植入物

無

標本送檢

- 細菌培養 無
- 細胞學 無
- 冰凍切片 無
- 病理標本
  組織名稱一： rectum
  ,
  組織名稱二： IMA root
- 截肢 無

標本交付 標本： 1 袋，標本： 0 盒，標本： 1 瓶，細胞學： 0 管，Culture Tube： 0 管，被交付醫師： R 蔡濬樟，

- 記錄者： 上次簽章: 劉雅汶
- 時間： 1540

傷口術後敷料

傷口敷料： 傷口敷料： 無

- 請於圖上標示部位：

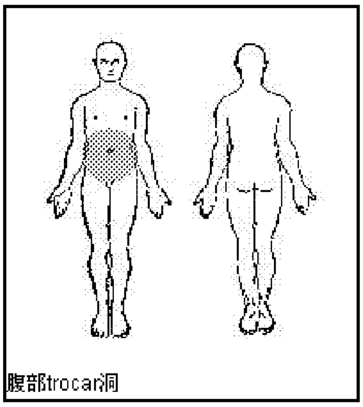

- 傷口敷料： mepore

導管

導管	天數	導管資訊	插入日期	插入時間	更換日期	拔除日期	拔除時間	拔除原因	預計拔除/更換	預計拔除/更換日期
CVC(1)	2	號碼:8 Fr; 深度:15cm; 部位:jugular (右); 象限:RU;	2021/11/18	0820						
Epidural PCA(1)	2	部位:硬脊膜;	2021/11/18	0800						
Foley(1)	2	號碼:16 Fr.; 部位:膀胱;	2021/11/18	0820						

用物計數

- 手術中出血量： minimal c.c.
- 手術中尿量： 350 c.c.
-
  - 其它： 無

用物計數：

- 部位一： 腹部，1.器械： 正確，2.縫針： 正確，3.刀片： 正確，4.紗布： 正確，5.腹部墊： 未使用，6.紗球： 未使用，7.PEANUTS： 未使用，8.紗條： 未使用，9.絲帶： 未使用，10.針頭： 未使用，11.Vessel loop： 未使用，12.Boots： 未使用，13.Jelco： 未使用，
  - 第一次：
    - 刷手護士 ： 林貝臻
    - 流動護士： 上次簽章: 廖子晴
    - 時間： 1420

  ,
  - 第二次：
    - 刷手護士 ： 林貝臻
    - 流動護士： 上次簽章: 廖子晴
    - 時間： 1430

  ,

- 第三次：
  - 刷手護士 ： 林貝臻
  - 流動護士： 上次簽章: 廖子晴
  - 時間： 1445
- 部位二： 直腸，1.器械： 正確，2.縫針： 正確，3.刀片： 正確，4.紗布： 正確，5.腹部墊： 未使用，6.紗球： 未使用，7.PEANUTS： 未使用，8.紗條： 未使用，9.絲帶： 未使用，10.針頭： 未使用，11.Vessel loop： 未使用，12.Boots： 未使用，13.Jelco： 未使用，14.棉條： 未使用，
  - 第一次：
    - 刷手護士 ： 歐真勤
    - 流動護士： 上次簽章: 廖子晴
    - 時間： 1410

  ,
  - 第二次：
    - 刷手護士 ： 歐真勤
    - 流動護士： 上次簽章: 廖子晴
    - 時間： 1415

  ,
  - 第三次：
    - 刷手護士 ： 歐真勤
    - 流動護士： 上次簽章: 廖子晴
    - 時間： 1420
-
  - 第一次：
    - 流動護士：

  ,
  - 第二次：
    - 流動護士：

  ,
  - 第三次：
    - 流動護士：

備註

備註： davinci 30度scopy:NO1

- 記錄者： 上次簽章: 歐真勤
- 時間： 0750

填寫護理人員 廖子晴

正式摘要

W52 / W52

doctorRank:B
